# Abnormal protein oligomers for neurodegeneration

**DOI:** 10.18632/oncotarget.18030

**Published:** 2017-05-19

**Authors:** Eiichi Tokuda, Yoshiaki Furukawa

**Affiliations:** Department of Chemistry, Keio University, Yokohama, Japan

**Keywords:** neurodegenerative disease, ALS, SOD1, protein misfolding, disulfide bond

Amyotrophic lateral sclerosis (ALS, also known as Lou Gehrig's disease) is a neurodegenerative disease that causes the death of motor neurons controlling voluntary muscle movement. After the appearance of early symptoms such as muscle weakness/stiffness, patients with ALS suffer from progressive muscular paralysis and usually die from respiratory failure within 2 to 5 years. Riluzole and Edaravone are the FDA-approved drugs that could prolong ALS survival; however, there is still no known effective cure/prevention for this devastating disease.

Most of the ALS cases are sporadic without any family histories, but an increasing number of genetic factors has been recently identified in the inherited form of the disease and contributes to our understanding on the pathomechanism of ALS [1]. Among those factors, *SOD1* encoding the protein, Cu/Zn-superoxide dismutase, is the first identified gene causative for inherited ALS [2]. Transgenic rodents expressing human SOD1 with mutations are well known to recapitulate the progressive and selective degeneration of motor neurons with ALS-like symptoms [3]. Experimental results *in vitro* and *in vivo* have hence been significantly accumulated on pathogenic roles of mutant SOD1 proteins, based upon which mutant SOD1 proteins are proposed to exert toxicities through their “misfolding” into the non-native conformations. Despite such extensive research on SOD1 and ALS, however, it still remains obscure how mutant SOD1 becomes misfolded in the pathological conditions *in vivo*.

**Figure 1:**
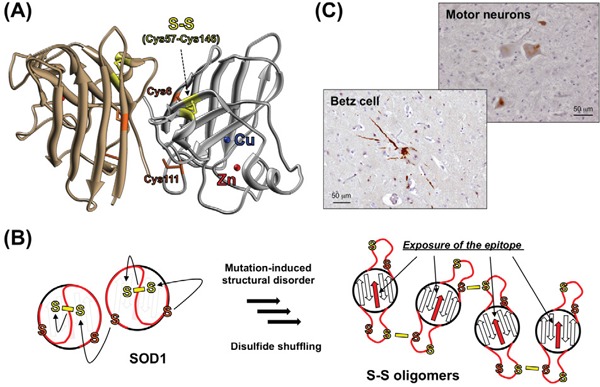
Formation of S-S oligomers in *SOD1*-related ALS **A.** Crystal structure of a natively folded SOD1 with copper (blue) and zinc (red) ions and an intramolecular disulfide bond between Cys57 and Cys146 (yellow) (PDB ID: 1HLA). Cys6 and Cys111 are also shown (orange). **B.** A disulfide-shuffling model for the formation of S-S oligomers. Cys6/111 (orange) attack Cys57/146 (yellow) forming the disulfide bond, which shuffles the disulfide bond among four Cys residues to form S-S oligomers. Upon formation of the S-S oligomers, the epitope for our anti-oligomer antibody (red arrows), which is buried in the folded conformation, becomes exposed and available for immunohistochemical examination. **C.** Immunohistochemical examination on Betz cells and spinal motor neurons in the ALS patients with SOD1 mutation (C111Y) using anti-oligomer antibody. Nuclei were also stained by hematoxylin (blue).

Native SOD1 binds copper and zinc ions and also forms an intramolecular disulfide bond (Figure 1A), and these post-translational factors bestow incredibly high structural stability on the natively folded conformation of the protein (*T*_m_ ~ 90 ^o^C). In contrast, ALS-causing mutations significantly destabilize the folded structure of SOD1 and facilitate its misfolding into non-native conformations [4, 5]. Among various non-native conformations that depend upon distinct experimental conditions, we have thus far proposed that the SOD1 oligomers cross-linked *via* disulfide bonds (called S-S oligomers) play pathological roles in *SOD1*-related ALS [6, 7].

SOD1 has four Cys residues of total, among which Cys57 and Cys146 usually form the intramolecular disulfide bond (Figure 1A). Mutant SOD1, in contrast, loses the natively folded structure at physiological temperatures (~37 ^o^C), which we have found facilitates the “shuffling” of the disulfide bond [4, 7]. Namely, the remaining two Cys residues (Cys6 and Cys111) are located away from the Cys57-Cys146 disulfide bond in the folded conformation, but the mutation-induced structural disorder allows Cys6/111 to nucleophilically attack either Cys57 or Cys146, resulting in the disulfide shuffling among the four Cys residues in SOD1 (Figure 1B). We have then considered that the S-S oligomers form when the disulfide shuffling occurs between SOD1 molecules. Formation of the S-S oligomers was well reproduced in a test tube, and also, we previously detected the S-S oligomers specifically in the spinal cords of symptomatic ALS model mice [6]. We thus attempted to further test if the S-S oligomers do form in the human *SOD1*-related ALS cases.

Unlike in the transgenic mice overexpressing mutant SOD1 proteins, nonetheless, it is expected to be difficult to biochemically characterize the S-S oligomers in human tissues, mainly because most of the spinal motor neurons in ALS patients are degenerated and usually absent at autopsies. In order to detect a trace amount, if any, of the S-S oligomers in ALS patients, therefore, we developed the anti-oligomer antibody that can specifically recognize the S-S oligomers. While detailed procedures for the antibody preparation were described in our original paper [8], the more extensive specificity test than ever before confirmed that our anti-oligomer antibody reacted only with the S-S oligomers but not the other states of SOD1 proteins *in vitro*. Then, ALS cases with a C111Y mutation in *SOD1* were examined with the anti-oligomer antibody, and motor neurons in spinal cord as well as Betz cells in motor cortex were selectively immunostained (Figure 1C). Furthermore, the spinal cord homogenates from the *SOD1*-related ALS patients produced signals with significant intensity in the sandwich ELISA using anti-oligomer antibody, but those signals disappeared upon pre-treatment of the homogenates with a reductant, dithiothreitol. Altogether, these results support our point that formation of the S-S oligomers is a pathological process in *SOD1*-related ALS.

Now that the S-S oligomers are considered as a pathological species in *SOD1*-related ALS cases, their toxicity toward motor neurons (*i.e.* pathogenicity) needs to be further examined, which is actually an on-going project in our group. Furthermore, many proteins other than SOD1 are also equipped both with disulfide bond(s) and free Cys residue(s); therefore, our disulfide-shuffling model may not be limited to the misfolding of SOD1 but could be one of the common mechanisms for protein misfolding *in vivo*.
